# Developing Intelligent Models to Detect and Classify Cattle Behavior on Pasture

**DOI:** 10.3390/ani16162617

**Published:** 2026-08-20

**Authors:** Alyssa Lopez, Elysia Jimenez, Damian Valles, Merritt L. Drewery

**Affiliations:** 1 Department of Agricultural Sciences, Texas State University, San Marcos, TX 78666, USA; 2 Ingram School of Engineering, Texas State University, San Marcos, TX 78666, USA

**Keywords:** animal welfare, artificial intelligence, behavior, cattle, computer vision, object detection, precision livestock farming, technology

## Abstract

Cattle producers must balance animal welfare with productivity, but regular observation of animals in extensively managed operations is often impractical. Artificial intelligence (AI) integrated with computer vision offers an automated alternative, but few studies compare object detection architectures within the same dataset or focus on pastured cattle. Groups (*n* = 2–7) of heterogeneous beef cattle were recorded on pasture with nine solar trail cameras. Footage (~132 h) was curated in VideoLAN; annotated in Computer Vision Annotation Tool (CVAT) with bounding boxes and behavioral classes; and split 62/21/17% into training (24,508 frames), validation (8231 frames), and testing (6625 frames) sets. Four architectures were trained: Faster R-CNN (ResNet-50 FPN), Single Shot MultiBox Detector (SSD300, VGG-16), RetinaNet (ResNet-50 FPN with focal loss), and YOLOv8 nano (Ultralytics). With validation at 0.50 confidence and 0.50 IoU, Faster R-CNN achieved the highest overall F1 (0.79) and best per-class balance; RetinaNet was intermediate (peak F1 = 0.72); SSD300 saturated at F1 = 0.40; and YOLOv8 nano achieved some minority class recall at lower confidence. Each model detected the classes “grazing” and “hay feeding” accurately but confused cattle with the visually similar “normal” class. Datasets, checkpoints, and analysis scripts are provided to support further refinement of AI-enabled monitoring of extensive cattle systems.

## Simple Summary

Cattle raised on pasture spend most of their time away from the producer. Therefore, when an animal is sick, stressed, and/or has poor welfare status, the condition could go undetected for days, delaying the necessary treatment and costing the producer more in associated costs. In this study, we assessed whether inexpensive trail cameras placed in a pasture, combined with computer software and a custom-developed model, could reliably recognize cattle and classify their behavior. We recorded 132 h of video of mixed herds of pastured cattle. Trained observers labeled short clips with the behavior displayed, and we used four computer vision programs to allow the model to learn from those labels. The best-performing program correctly recognized common behaviors–such as grazing and consuming hay from a feeder–more than 80% of the time but struggled with less common behaviors, such as fear or dominance interactions. We have shared the dataset, performance, and analytical code to allow other researchers to improve on the model described here. User-friendly automatic monitoring of pastured cattle could help producers identify problems sooner and improve animal welfare, promoting the sustainability of beef and dairy production.

## 1. Introduction

Increased demand for animal-source products has pressured the livestock sector to increase output and efficiency [1]. To address these demands, agricultural producers are incorporating novel technologies into their operations to facilitate routine functions [2] that would otherwise require more time and labor [3]. Due to the expanding capabilities of artificial intelligence (AI), implementing AI-enabled technologies that monitor the behavior of agricultural animals may be viable for producers [4].

It is well established that stress and certain handling practices negatively affect animal health and performance [5,6,7,8] and, thus, reduce efficiency and profitability. The level of animal welfare associated with animal agriculture is becoming increasingly important to consumers [9,10], influencing purchasing decisions in favor of products that consumers perceive as benefiting animals and the environment [9,10,11,12]. In response, livestock management practices are increasingly focused on enhancing animal welfare, productivity, and efficiency by adopting mindful handling procedures and implementing computer-based technologies [1,13].

Animal welfare status can be evaluated through observation [14] as animals often reflect how well their needs are met in their behavior and physiology [15]. However, relying on manual observation requires significant time, labor, and training to identify signs of decline in an animal’s condition [13,14,16,17,18]. Further, manual observation in extensive operations is impractical due to the growing scale of modern ranches [19,20], which can allow disease progression and other welfare concerns to go unnoticed without timely intervention [14,18].

Production goals and resource availability vary across cattle industry sectors [21], but productivity losses associated with welfare issues are similar [22,23,24,25]. Pasture-based systems face additional challenges compared with intensive, confined systems, as the remoteness and environmental variability complicate access to animals [26]. As a result, challenges and losses in pasture-based systems may be preventable by integrating technologies like those used in intensively managed systems [22,23,24,25,27].

Recently, computer vision systems have been combined with AI to record behavioral patterns and analyze positional changes in cattle over time [28]. These systems have the potential to provide results comparable to those of devices that monitor cattle behavior and health on an individual-animal basis [1], such as collars or other wearable devices [29]. Establishing a system to detect “normal” behavior in cattle can facilitate the detection of variations from baseline conditions in individuals and herds. These AI-enabled systems are often touchless and automated, reducing the need for additional animal handling, which can promote positive animal welfare and worker safety.

The foundation of any computer vision system for cattle monitoring is the underlying object detection system, which must reliably localize each animal within a frame and assign the correct class label before any downstream inference can be made. Modern deep learning detectors fall into two broad families: two-stage detectors, which first generate candidate regions and then classify them, and single-stage detectors, which predict class labels and bounding box coordinates simultaneously in a dense, pixel-wise manner. Each family of detectors offers distinct trade-offs between detection accuracy, inference speed, and robustness to scale variation, occlusion, and class imbalance, all of which are characteristic of imagery captured from extensively managed cattle systems. To investigate which architectural family is most suitable for pastured cattle, the present study compares four widely adopted detectors: Faster R-CNN [30], Single Shot MultiBox Detector (SSD) [31], RetinaNet [32], and the eighth generation of the You Only Look Once detector (YOLOv8) [33,34].

Faster R-CNN [30] is a canonical two-stage object detector; it introduces a Region Proposal Network (RPN) that shares convolutional features with downstream classification and bounding box regression heads, enabling end-to-end training and near-real-time inference with high accuracy. Because only a few hundred high-quality proposals are passed to the classifier, Faster R-CNN often delivers superior localization accuracy on objects with complex shapes and varying scales [30], which is desirable when footage is captured at varying distances from a fixed camera and against cluttered backgrounds (e.g., bushes, trees, fences). Faster R-CNN and its instance-segmentation extension, Mask R-CNN, have been applied to cattle in precision livestock contexts, including pixel-accurate segmentation and contour extraction of grazing cattle for body condition scoring and behavior analysis [35], visual identification of individual dairy cows via deep metric learning over Faster R-CNN region features [36], and aerial classification and counting of animals from quadcopter imagery [37]. Comparative studies of detector families using ecological camera trap data have also reported strong performance by Faster R-CNN on free-ranging animals across heterogeneous backgrounds [38]. Collectively, these data indicate that the Faster R-CNN architecture is well suited to detect cattle in scenarios in which accuracy is prioritized over inference speed.

The SSD [31] is a single-stage detector that predicts bounding boxes and class scores from multiple feature maps of different spatial resolutions in a single forward pass. The use of multi-scale feature maps allows SSD to detect both large foreground animals and smaller, more distant animals using the same network, without the computational overhead of a RPN stage [31]. This property is particularly relevant to pasture-based imagery, where individual cattle may occupy only a small fraction of the frame when far from the camera but appear large and partially occluded near the camera. SSD-style multi-scale, single-shot detection has been adopted in livestock and wildlife studies for detection and counting in unmanned aerial vehicle imagery, consistently demonstrating a favorable balance between speed and detection accuracy on animal subjects in heterogeneous environments [39]. The same multi-scale principle has further been used as a baseline in deep-learning pipelines for ecological camera trap data [38], reinforcing the suitability of SSD-class detectors for free-ranging animals captured at variable distances and viewpoints (e.g., camera height).

RetinaNet [32] is a single-stage detector built on a Feature Pyramid Network (FPN) backbone and is best known for introducing focal loss, which is a reshaping of the standard cross-entropy loss that down-weights well-classified classes and focuses training on difficult or less common, misclassified classes. This explicitly addresses the extreme foreground–background class imbalance that arises in dense, anchor-based detection, allowing single-stage detectors to match or exceed the accuracy of two-stage detectors while also retaining their speed [32].

For pastured cattle, where candidate boxes likely correspond with grass, sky, or vegetation rather than animals, focal loss is expected to mitigate the bias toward “background” predictions that have been observed in standard detectors. The combination of FPN-based multi-scale features and focal loss positions RetinaNet as a strong baseline for animal detection tasks involving small, dense, or partially occluded subjects, and the focal loss principle has subsequently been adopted across detector variants used in cattle and broader animal phenotyping [32,40].

YOLO [33] is a single-stage detector that frames object detection as a single regression task from image pixels to bounding box coordinates and class probabilities. The unified architecture of YOLO enables real-time inference, which is critical when continuously processing many hours of footage. YOLOv8 [34], the most recent version available at the time of this experiment, incorporates an anchor-free decoupled head, an updated Cross Stage Partial (CSP)-based backbone, and refined data augmentation strategies, yielding strong accuracy with compact model sizes.

The YOLO family has been a widely adopted object detector in cattle and broader animal agriculture research, with documented use cases including hierarchical cattle behavior recognition with spatio-temporal information [28], non-destructive identification and tracking of multi-object behaviors in beef cattle [41], YOLO-BoT-based behavior recognition and multi-object tracking [42], a recent review of YOLO algorithms in animal phenotyping applications [40], and detection and tracking of free-ranging animals in field environments [43]. Together, these studies establish YOLO and, specifically, YOLOv8 as a practical, high-throughput baseline against which two-stage and dense single-stage detectors can be compared on the same dataset.

Although research on pastured cattle is growing, AI-enabled technologies that detect and classify animal behavior have primarily been developed for and applied to intensive operations, although significant production losses are related to animal welfare in extensive systems [2,22,23,24]. Research utilizing intelligent systems to enhance animal welfare and production efficiency in extensive cattle operations remains limited, and current studies do not predict animal behavior on pasture, nor do they systematically compare various detection backbones on the same dataset. Accordingly, the objective of our study was to develop, validate, and test an AI-enabled model built on four object detection architectures (i.e., Faster R-CNN, SSD, RetinaNet, and YOLOv8 nano), to detect pastured cattle and predict their behavior.

## 2. Materials and Methods

### 2.1. Overview

**Figure 1:**
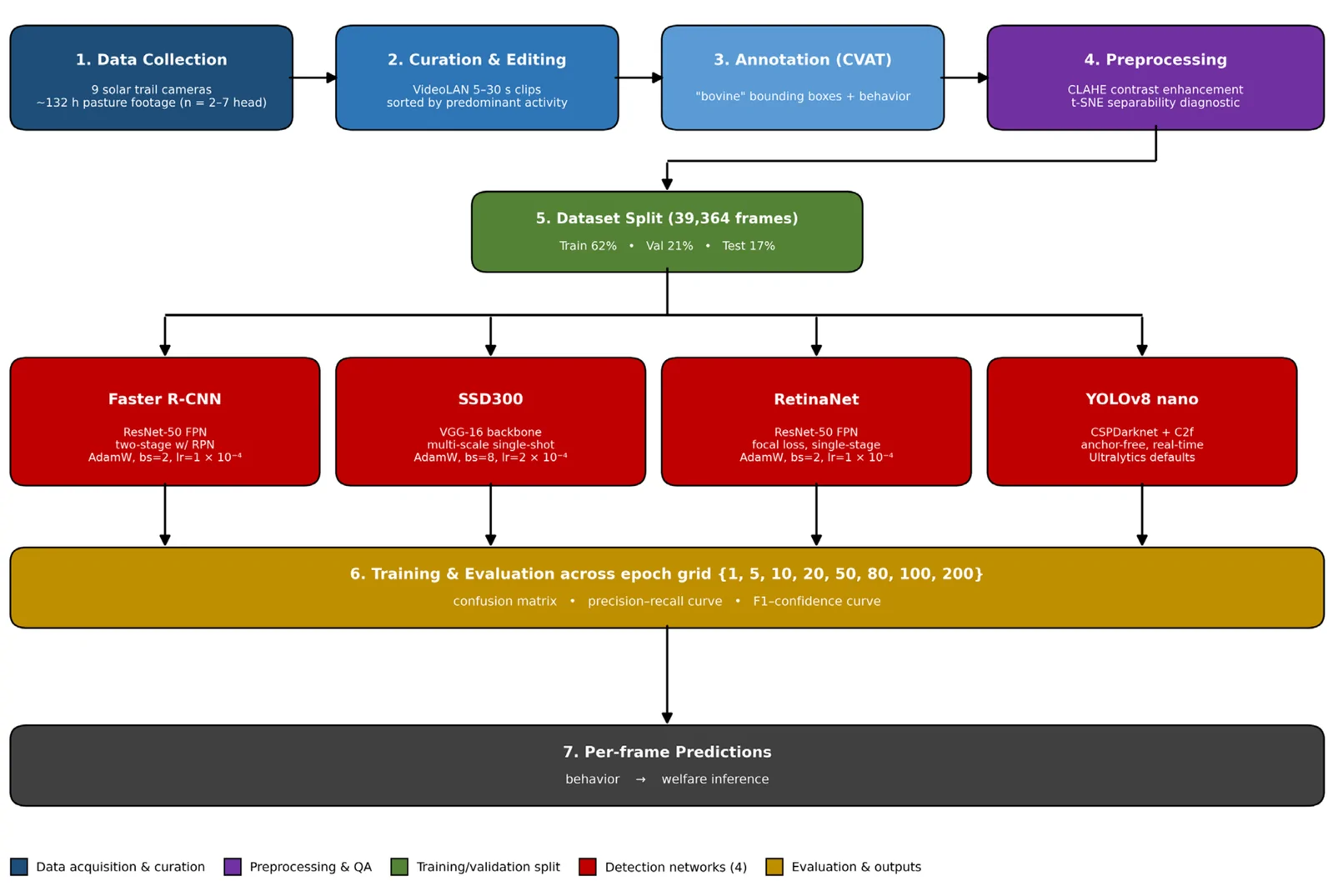
Overview of the cattle detection and behavior prediction pipeline implemented in this study.

An overview of the study workflow is presented in Figure 1. Continuous recordings from nine cameras were curated into short clips, annotated in Computer Vision Annotation Tool (CVAT) with cattle bounding boxes and behavior classes, and preprocessed with Contrast Limited Adaptive Histogram Equalization (CLAHE) and a t-distributed Stochastic Neighbor Embedding (t-SNE) separability diagnostic before being partitioned into training, validation, and testing splits. The same splits were then used to train and evaluate four detection architectures (i.e., Faster R-CNN, SSD300, RetinaNet, and YOLOv8 nano) across a common epoch grid, producing per-class confusion matrices, precision–recall curves, and F1–confidence curves that underlie the per-frame predictions of cattle behavior.

### 2.2. Data Collection

These procedures were approved by the Institutional Animal Care and Use Committee (#9298) at Texas State University. Cattle were sourced from the commercial herd at a university-operated ranch (Freeman Center, Texas State University). The university maintains a commercial herd for educational and research purposes. Groups (*n*~2–7) of cattle were visually recorded on pasture using nine solar trail cameras (Voopeak, CA, USA; 120° field of view, 1080p, 30 frames/s) placed in stationary positions at Freeman Center in San Marcos, TX (29.93827° N, −98.00834° W). Cattle were phenotypically and physiologically heterogeneous (e.g., bulls, cannulated steers, cows, calves) to capture inter-animal variation in characteristics. The trial was conducted over three months with continuous recording during group rotations, which occurred for 12–24 h every 2–3 weeks, to capture a varied environment.

### 2.3. Data Curation and Editing

Raw footage (~132 h) was stored on secure digital (SD) cards. Footage containing cattle in the frame was clipped and edited with VideoLAN version 3.0.20. Clips of cattle exhibiting specific behavior for five to thirty seconds were curated and filed in digital subfolders with the corresponding label.

### 2.4. Data Annotation

Videos were manually annotated using the program CVAT. Individual cattle were labeled as “bovine” and identified with bounding boxes. Specific behavioral classes were added to the bovine label, as outlined in Table 1.

Annotation was performed by three trained observers using a shared behavior-definition sheet adapted from established cattle-ethogram references. The sheet was calibrated on a common set of 200 training clips before independent labeling began; a fourth reviewer audited 10% of the labels for consistency. Because the “normal” and “fear response” classes are relatively subjective, we will report inter-observer agreement (Cohen’s κ and Krippendorff’s α) on a stratified 10% resample in the next release of the dataset and will re-define or merge any class with κ < 0.60.

### 2.5. Contrast Limited Adaptive Histogram Equalization (CLAHE)

Collected images were processed to improve contrast and differentiate each animal from the environment, which can introduce false-positive detections due to information clarity, even when individual animals are annotated with bounding coordinates. Therefore, CLAHE was applied to the training dataset to enhance contrast and features, enabling the model to detect more clearly with fewer epochs. Enhancement via CLAHE helps the model detect details when animals are obstructed or further from the camera or when the environment is unfavorable (e.g., overcast weather). CLAHE was applied only to the training split; validation and test frames were used at their native contrast so that reported validation and test-set metrics reflect the model on unmodified, in-the-wild imagery.

**Figure 2:**
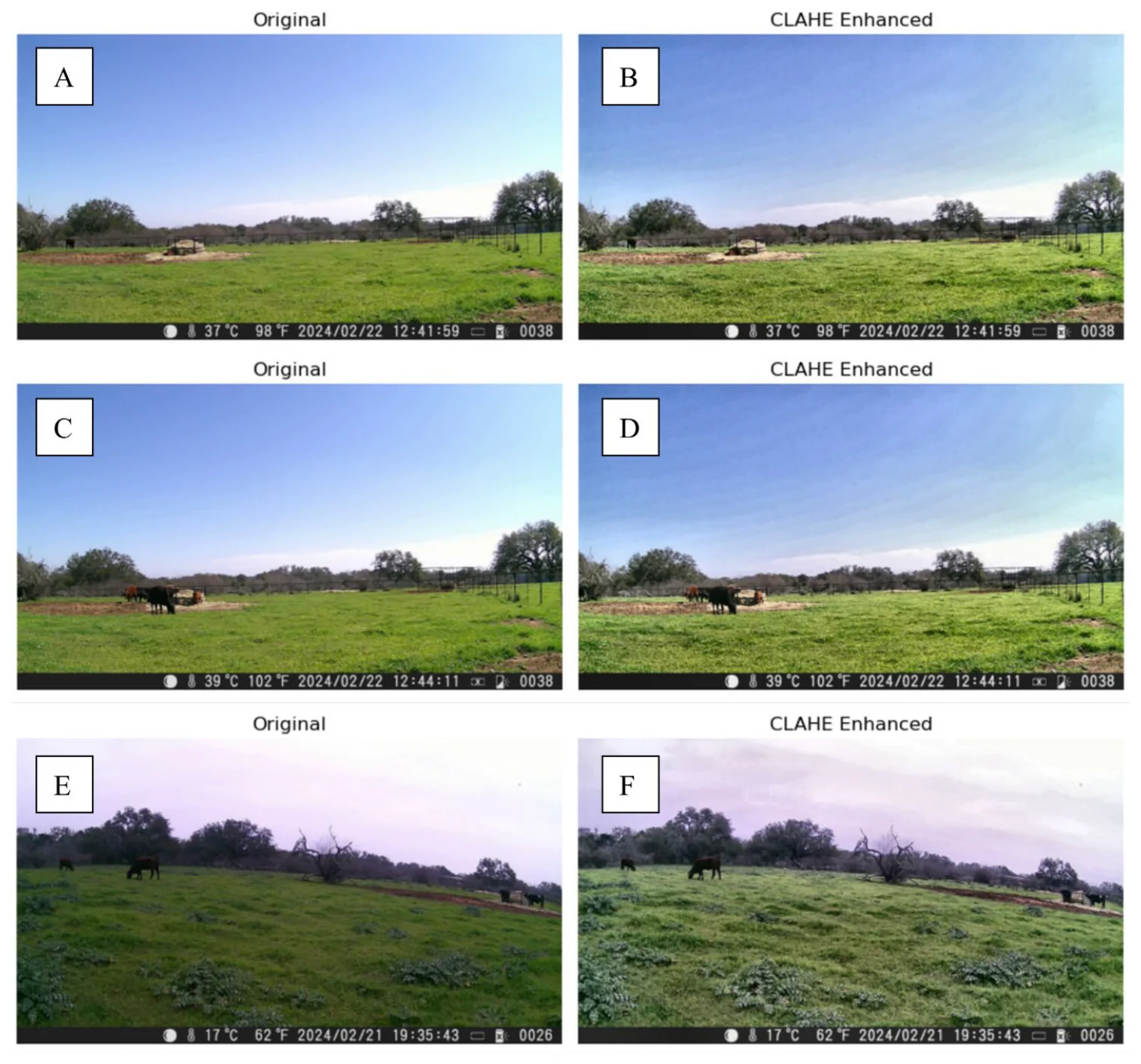
Examples of Contrast Limited Adaptive Histogram Equalization (CLAHE) applied to various frames to enhance model performance in detecting pastured cattle and predicting their behavior. (**A**,**B**) comparison of CLAHE on animals far from the camera; (**C**,**D**) comparison of CLAHE on a clustered herd; (**E**,**F**) comparison of CLAHE on a darker image with grazing cattle.

Figure 2A,B display the contrast enhancement achieved by CLAHE on an RGB image of the pasture in the training split; there is greater contrast between the grass and the darker background of the trees. The single animal in the image can be more easily identified than its background, given the distance from the camera. Figure 2C,D depict a cluttered herd in one section of the image frame. The contrast enhancement more precisely separates cattle and their features; cattle in the foreground can be separated from the background, and the remaining animals are behind the frame. In the background, each animal is more distinct in its features, providing clarity for the model to predict behaviors even when individual animals are close to other objects or animals. Figure 2E,F are further examples of CLAHE enhancement, depicting a clearer foreground, animals, and separation of the trees in the background. The enhancement provides clearer features of the cattle on the left side of the frame; however, it is still difficult to determine objects and activities on the right side of the frame.

### 2.6. Feature Extraction and t-SNE

To examine the feature representations learned by deep convolutional backbones for each class and assess the ability of each detector head to predict the correct class, a feature extraction and dimensionality-reduction workflow was applied to a stratified subsample of the training data split. After bounding boxes were generated by the YOLOv8 nano detector during the curation stage, each cropped region was passed through a convolutional backbone consistent with those used by the four detectors evaluated in this study (i.e., Faster R-CNN, SSD, RetinaNet, and YOLOv8), and global-pooled penultimate-layer activations were retained. This produced a high-dimensional embedding of *n* = 3000 crops × 2048 features in which each crop is represented by a single non-negative feature vector summarizing its visual content. Features used for the t-SNE analysis were the global-pooled 2048-dimensional penultimate-layer activations of a COCO-pretrained ResNet-50 applied to bounding box crops produced by the YOLOv8 nano detector during the curation stage (Section 2.3). Each crop was paired with the predominant behavior label assigned by the annotators in Section 2.4.

Each crop was paired with the predominant behavior class assigned during annotation, yielding five classes: “grazing” (*n* = 1221; 40.7%), “hay feeding” (*n* = 1211; 40.4%), “normal” (*n* = 518; 17.3%), “dominance assertion” (*n* = 32; 1.1%), and “fear response” (*n* = 18; 0.6%); other classes <1% as described in Table 1 were not represented in the data split at a high enough frequency for inclusion. The strong class imbalance present in this subsample mirrors the natural time budget of pastured cattle [44] and was preserved across the training, validation, and testing splits used by each architecture.

The 2048-dimensional feature space cannot be visualized directly. To inspect the class structure, the extracted features were projected into two- and three-dimensions using t-SNE [45]. t-SNE is a non-linear, neighborhood-preserving dimensionality reduction technique that converts pairwise distances in the high-dimensional space into conditional probabilities expressing the likelihood that one point would select another as its neighbor under a Gaussian kernel, and then seeks a low-dimensional embedding whose pairwise probabilities, modeled with a heavier-tailed Student’s t-distribution, match the high-dimensional ones as closely as possible by minimizing Kullback–Leibler divergence between the two distributions [45]. Because t-SNE prioritizes the preservation of local neighborhoods over global Euclidean distances, it is well suited to reveal fine-grained cluster structure in deep network feature spaces in which the global geometry is highly non-linear. This technique was diagnostic in the current study; it was used to assess class separability in the representations used to train the detectors and not for prediction.

**Figure 3:**
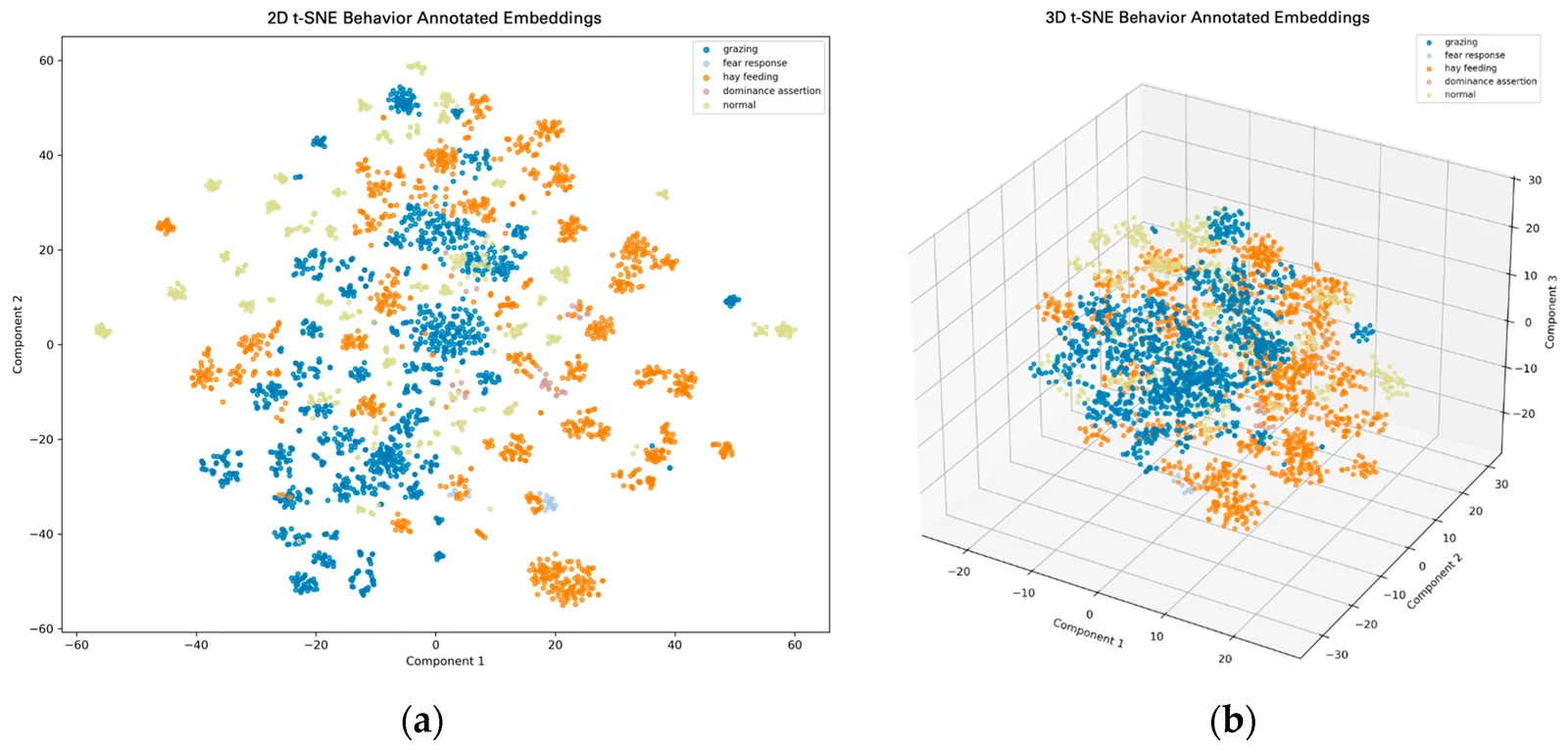
t-distributed Stochastic Neighbor Embedding (t-SNE) features from the annotated behavior classes: (**a**) 2D embedding. (**b**) 3D embedding.

The resulting two-dimensional and three-dimensional embeddings are displayed in Figure 3a,b, with each point colored by its annotated behavior. Three observations from the embeddings directly informed the interpretation of the detector results that follow. First, the two majority classes (i.e., “grazing” and “hay feeding”) formed numerous well-defined sub-clusters, but those sub-clusters are interleaved across the projection, with “grazing” and “hay feeding” points often appearing as adjacent or partially overlapping regions rather than as a single contiguous cluster per class. This sub-cluster structure reflects multiple visual sub-types of the same behavior (e.g., different camera-to-animal distances, animal orientations, lighting conditions) and yields locally pure neighborhoods that are, nevertheless, globally fragmented. Second, the “normal” class occupied an intermediate region that overlaps both “grazing” and “hay feeding”, consistent with its definition as a non-specific resting or standing state that visually resembles low-activity grazing or non-feeding posture at a feed bunk. Third, the behaviors represented to a lower extent in our dataset (i.e., “fear response” and “dominance assertion”) appeared as small, isolated pockets embedded within larger “grazing” and “hay feeding” regions, with too few example images to form a coherent class boundary.

The t-SNE analysis provides a representation-level explanation for the per-class accuracy and confusion and motivates the comparison of four architectures we conducted in this work: each network’s inductive biases interact differently with the cluster structure documented here, and no single detector is expected to be uniformly “best” across all behavior classes.

### 2.7. Detection and Classification Using Deep Learning

Four detection architectures were compared under a common training and evaluation protocol such that any performance differences could be attributed to the architecture rather than dataset preparation or optimization choices. All four models consumed the same YOLO-formatted images and label files described in Section 2.4 (Table 1), and identical training, validation, and testing splits (24,508/8231/6625 frames; 62/21/17%). The three PyTorch-based detectors (i.e., Faster R-CNN, SSD300, and RetinaNet) were implemented with a torchvision.models.detection module using COCO-pretrained weights (weights = “DEFAULT”) and their classification heads were replaced to output the C+1 classes required by the current dataset (C behavior classes plus the implicit background class). YOLOv8 nano was trained with Ultralytics implementation using published default hyperparameters for the YOLOv8n.pt detection model. To ensure reproducibility, every training run was initialized with a fixed random seed (seed = 42) and used the same DataLoader collate function for detection targets.

#### 2.7.1. Training Considerations

The three torchvision detectors share an identical training loop implemented in a common helper module (referred to internally as detector_common). For each detector, the training loop performs the following per epoch: (1) a full pass over the training split under AdamW optimization; (2) full-split validation reporting precision, recall, F1, true-positive, false-positive, and false-negative counts at a fixed intersection over union (IoU) threshold of 0.50 and a score threshold of 0.50; (3) a StepLR schedule that decays the learning rate by a factor of 0.10 every 5 epochs (step_size = 5, γ = 0.10); and (4) checkpointing of the latest weights plus the best F1 weights observed to that point. Post-training analysis (per-class precision, recall, and confusion matrices) is executed with the same helper. This design isolates network architecture as the only meaningful variable between the torchvision runs.

The dataset was partitioned at the clip level: each 5–30 s edited clip produced in Section 2.3 was assigned in its entirety to exactly one split, so no two frames derived from the same continuous video segment appear in different splits. The resulting partition contained 473 training clips (24,508 frames; 62%), 158 validation clips (8231 frames; 21%), and 128 testing clips (6625 frames; 17%). Frames from consecutive clips within the same recording day were also kept in the same split whenever cattle identity was constant, so residual frame-level similarity across splits was minimized. All four detectors were trained on the identical class list defined in CMB_config.yaml, so per-model differences are not attributable to differences in the label set. Classes with fewer than approximately 100 training exemplars (fear response, dominance assertion, grooming, vocalizing, sniffing) provided essentially no learning signal and were not reliably predicted by any of the four architectures.

#### 2.7.2. Faster R-CNN Specifications

Faster R-CNN was instantiated as torchvision.models.detection.fasterrcnn_ resnet50_fpn(weights = “DEFAULT”) and the FastRCNNPredictor head was replaced to output the required number of classes. The ResNet-50 + FPN backbone produced multi-scale proposals via the built-in RPN, and each proposal was refined by the two-stage classification and bounding box regression head. Training used a per-device batch size of 2 images for 20 epochs, with an initial learning rate of 1 × 10^−4^ and weight decay of 1 × 10^−4^ (AdamW). This configuration reflects the higher per-image memory footprint of two-stage detectors relative to single-stage networks and prioritizes localization accuracy over throughput, in line with the architectural rationale outlined above.

#### 2.7.3. SSD300 Specifications

SSD300 was instantiated as torchvision.models.detection.ssd300_vgg16 (weights = “DEFAULT”) with the SSDHead replaced to match the number of classes and the built-in default anchor generator retained (i.e., the number of anchors per feature map location supplied to the new head was queried directly from the pretrained model’s anchor generator). All input frames were resized to 300 × 300 pixels, the resolution required by the pretrained backbone before batching. Training used a batch size of 8 images for 20 epochs, an initial learning rate of 2 × 10^−4^, weight decay of 1 × 10^−4^ (AdamW), the same StepLR schedule, and 0.50 score/IoU thresholds as the other torchvision detectors. The larger batch size and slightly higher learning rate reflect SSD’s single-stage, forward-only computation and its lower per-image memory footprint after resizing.

#### 2.7.4. RetinaNet Specifications

The RetinaNet was instantiated as torchvision.models.detection.retinanet_resnet50_fpn(weights = “DEFAULT”), and the classification and regression subnets were replaced by a new RetinaNetHead configured for the current number of classes and the number of anchors per location inherited from the pretrained head. The FPN backbone was retained, preserving the P3–P7 pyramid on which RetinaNet’s dense prediction relies, and the built-in focal loss (γ = 2.0, α = 0.25 in the torchvision implementation) was used without modification. Training matched Faster R-CNN’s per-image budget with a batch size of 2 images for 20 epochs, AdamW with an initial learning rate of 1 × 10^−4^ and weight decay of 1 × 10^−4^, StepLR (step_size = 5, γ = 0.10), and an identical 0.50 score/IoU threshold during evaluation.

#### 2.7.5. YOLOv8 Nano Specifications

The YOLOv8 nano (YOLOv8n.pt) was trained using the current published default hyperparameters of the Ultralytics implementation to enable direct comparison with prior work reporting YOLOv8 results “out of the box.” YOLOv8n is a compact, anchor-free, single-stage detector composed of a CSPDarknet backbone with C2f modules, an SPPF neck, and a decoupled detection head that predicts objectness, class logits, and distribution focal loss (DFL) offsets.

Under Ultralytics defaults, training used an input image size of 640 × 640 pixels, a batch size of 16 images, and up to 100 epochs with an early-stop patience of 100. Optimizer selection was set to optimizer = “auto”, which chooses SGD for YOLOv8-class models with initial learning rate (lr0) = 0.01, final-LR fraction (lrf) = 0.01, momentum 0.937, and weight decay 5 × 10^−4^. The first three epochs were warmup epochs during which momentum ramped from 0.8, and bias learning rate ramped from 0.10.

The composite training loss weights were box = 7.5, cls = 0.5, and DFL = 1.5, and automatic mixed precision (AMP) training was enabled by default. Non-maximum-suppression IoU during validation was 0.70, and the default confidence threshold at prediction time was 0.25 (0.001 for validation reporting). Photometric and geometric augmentation followed Ultralytics defaults (HSV-H = 0.015, HSV-S = 0.70, HSV-V = 0.40; translate = 0.10; scale = 0.50; horizontal flip probability 0.50), together with mosaic = 1.0 for all but the final 10 epochs (close_mosaic = 10), mixup = 0.0, and copy-paste = 0.0. To keep the epoch grid comparable across networks, YOLOv8 nano was also evaluated at the same {1, 5, 10, 20, 50, 80, 100, 200} epoch checkpoints as described below.

A summary of the models’ hyperparameters is provided in Table 2. AdamW defaults were β_1_ = 0.9, β_2_ = 0.999, and ε = 1 × 10^−8^, as used by torchvision’s built-in AdamW instantiation; these were not overridden in the attached scripts.

Each of the four architectures was trained across a common epoch grid of 1, 5, 10, 20, 50, 80, 100, and 200 epochs. At each checkpoint, a confusion matrix, a precision–recall curve, and an F1–confidence curve were generated on the validation split to identify the optimal training length for each network independently. Confusion matrices reported true class along the *y*-axis and predicted class along the *x*-axis, allowing per-class analysis of misclassifications; precision–recall curves quantified each architecture’s ability to detect an animal in the frame and correctly predict its behavioral class (Equations (1) and (2)); and F1–confidence curves reported the harmonic mean of precision and recall as a function of the model’s minimum confidence threshold for a positive prediction (Equation (3)). Based on training and validation performance, the behavior model was tested at 5, 10, and 20 epochs; the same three-epoch grid was applied to each of the four detectors so that per-network results are directly comparable.

(1) $$Precision\;=\;\frac{True\;Positive}{True\;Positive\;+\;False\;Positive}$$

(2) $$Recall\;=\;\frac{True\;Positive}{True\;Positive\;+\;False\;Negative}$$

(3) $$F1\;Score=\;2\;\times \;\frac{Precision\;\times \;Recall}{Precision\;+\;Recall}$$

## 3. Results

The four detection architectures were evaluated on the same validation split at a confidence threshold of 0.50 and an IoU threshold of 0.50. Training and validation loss curves for each network indicated that bounding box loss on the validation split decreased until approximately 20 epochs before rising, whereas class loss rose more sharply beyond 20 epochs, indicating 10–20 epochs as the effective training window for the two majority classes (i.e., grazing and hay feeding). Best-F1 checkpoints written during training were therefore used for all cross-model comparisons.

Overall validation performance is summarized in Table 3. Faster R-CNN achieved the highest overall F1 (0.79) and best balance between precision (0.75) and recall (0.83). RetinaNet was intermediate, with a peak validation F1 of 0.72 at a confidence threshold of 0.75 (F1 0.70 at 0.50 confidence). SSD300 saturated at a lower peak F1 of 0.40 at low confidence (0.25) and did not recover with tighter thresholds (F1 0.39 at 0.50 confidence). YOLOv8 nano results achieved the lowest overall F1 (0.27) and poorest balance between precision (0.25) and recall (0.25).

### 3.1. YOLOv8 Nano

**Figure 4:**
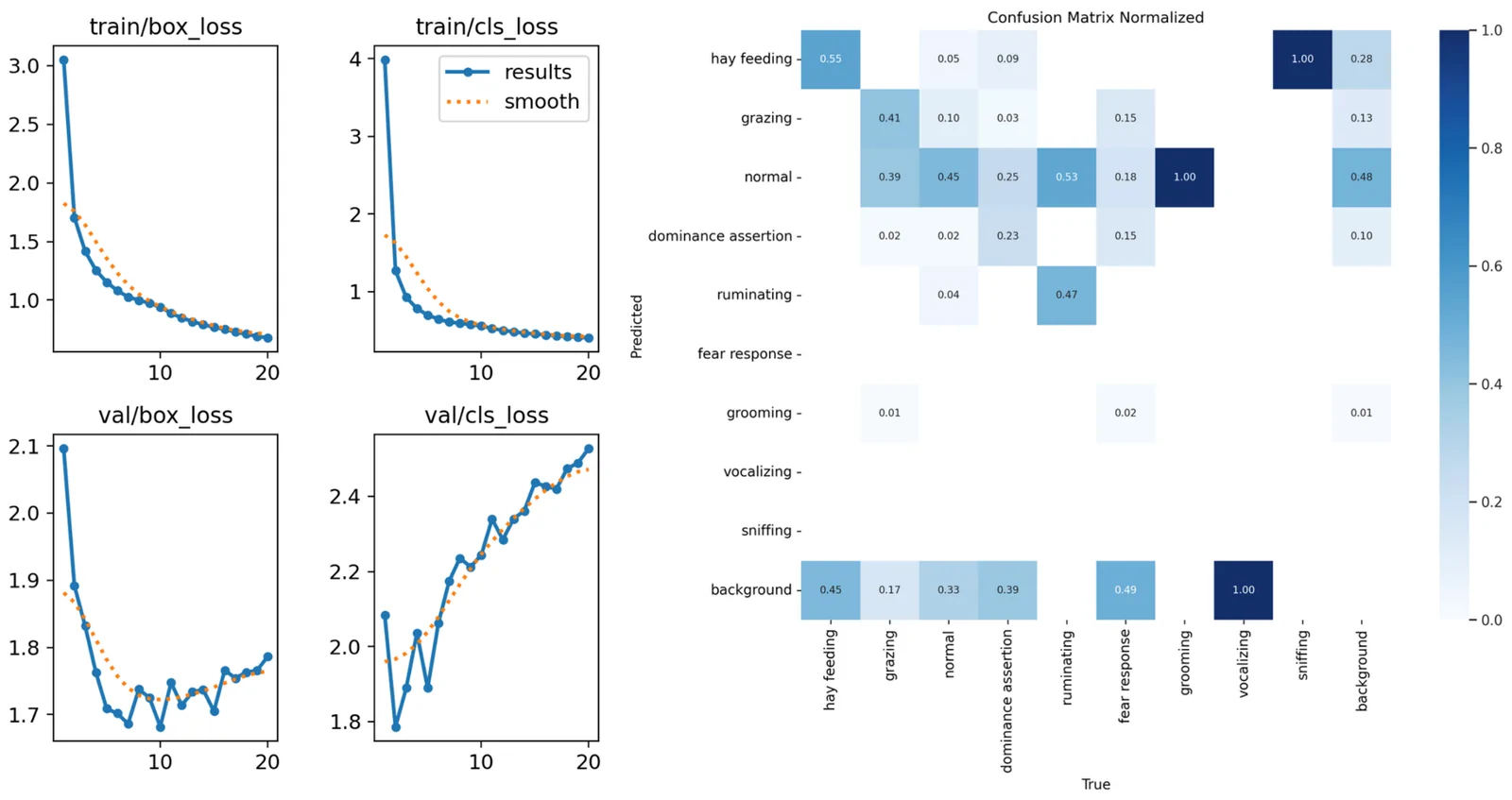
Performance of the cattle behavior model after 20 epochs with early stopping.

Performance of the behavior model after 20 epochs (with early stopping) is displayed in Figure 4. The upper graphs display performance for training segments, and the lower graphs display performance for validation segments. Loss associated with the bounding box (i.e., animal detection) is displayed on the left, and loss associated with the predicted behavior is displayed on the right. As the model was analyzed over a progressively increasing number of epochs, bounding box loss on the validation segments decreased until the number of epochs surpassed 20, then increased, indicating 20 epochs as the maximum. The validation class loss increased sharply with increasing epochs; accordingly, analyzing the model at 10 epochs provides a potential compromise between the box and class losses. Therefore, the number of epochs to optimize model performance ranged from 10 to 20.

“Hay feeding” was correctly predicted (i.e., true positives) in 55% of frames, “grazing” in 41%, “normal” in 45%, “dominance assertion” in 23%, and “ruminating” in 47%, while “fear response”, “grooming”, “vocalizing”, and “sniffing” were not predicted in any frames (Figure 4). The “background” class denotes image regions that contain no labeled object of interest; detection models learn to distinguish these regions from true objects, because recognizing “nothing” is part of the computational budget.

**Figure 5:**
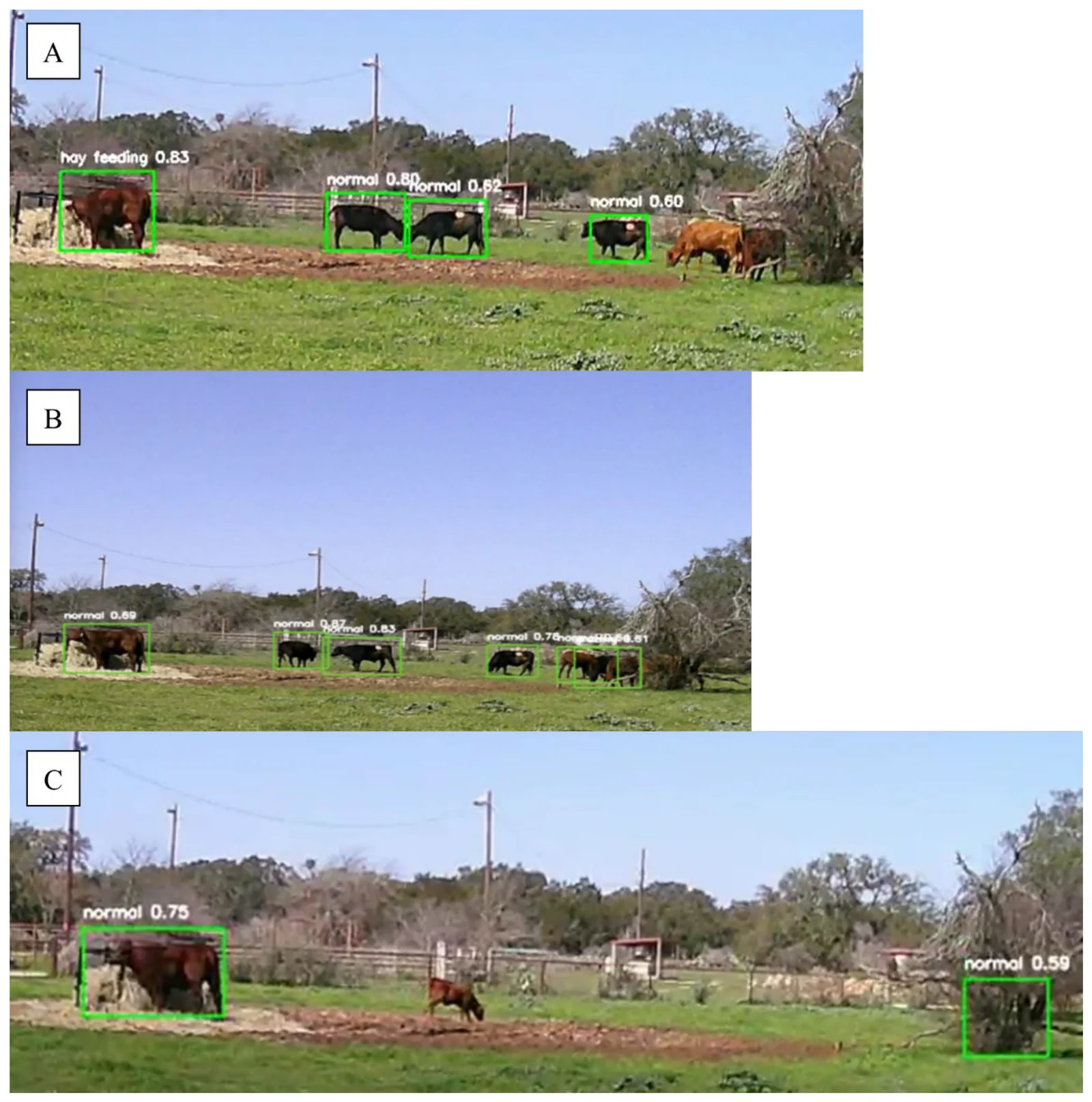
Performance of the testing split of an artificial intelligence (AI)-enabled computer vision model developed to classify and predict pastured cattle behavior at 20 (panel (**A**)) and 10 (panels (**B**,**C**)) epochs.

The predicted behavioral classes demonstrated lower true-positive rates and confidence levels at 20 epochs. Testing the model at lower epochs increased cattle detection in the frames but also led to more misdetections due to heterogeneous environmental variables (e.g., lighting, vegetation, structures, and equipment). Based on the confusion matrix, at 20 epochs, the model predicted multiple behaviors as “background,” indicating that cattle in the frame were not detected as “bovines” and their behavior was not predicted. Example predictions from YOLOv8 nano at 10 and 20 epochs are provided in Figure 5.

### 3.2. Faster R-CNN

**Figure 6:**
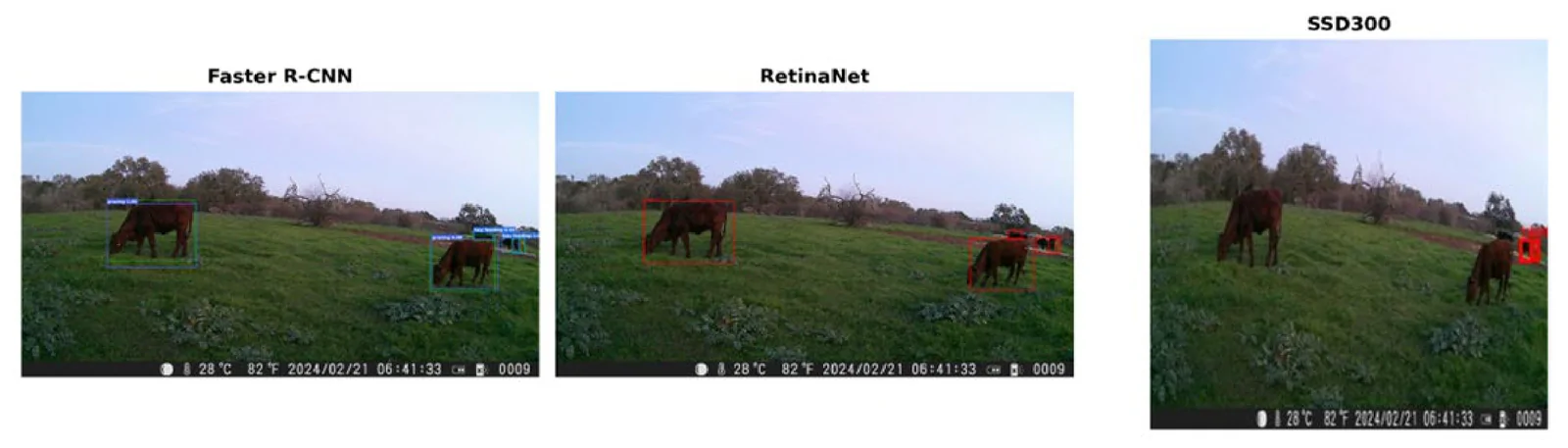
Same validation frame (21 February 2024 06:41:33) processed by Faster R-CNN, RetinaNet, and SSD300.

Faster R-CNN delivered the highest overall validation F1 (0.79 at 0.50 confidence) across the four architectures (Table 3); an example of the same validation frame processed by Faster R-CNN, RetinaNet, and SSD300 is provided in Figure 6.

At a score threshold of 0.50 and an IoU threshold of 0.50 (aggregated across the classes “grazing”, “hay feeding”, and “normal”), the model produced 9003 true positives, 3077 false positives (“background” predicted as one of the three classes), and 1847 false negatives (annotated animals missed or mislocalized), for an overall precision of 0.75, recall of 0.83, and F1 of 0.79.

Per-class performance (Table 4) was strongest for “hay feeding” (recall 0.91, precision 0.84, F1 0.87), intermediate for “normal” (recall 0.81, precision 0.63, F1 0.71), and weakest for “grazing” (recall 0.78, precision 0.57, F1 0.66); the decline in precision for the “grazing” class was driven by confusing “grazing” with the “normal” class at close distance (795 “grazing” labels predicted as “normal” and 677 “normal” labels predicted as “grazing”). Class counts by split (ground-truth annotations) are provided in Table A1.

**Figure 7:**
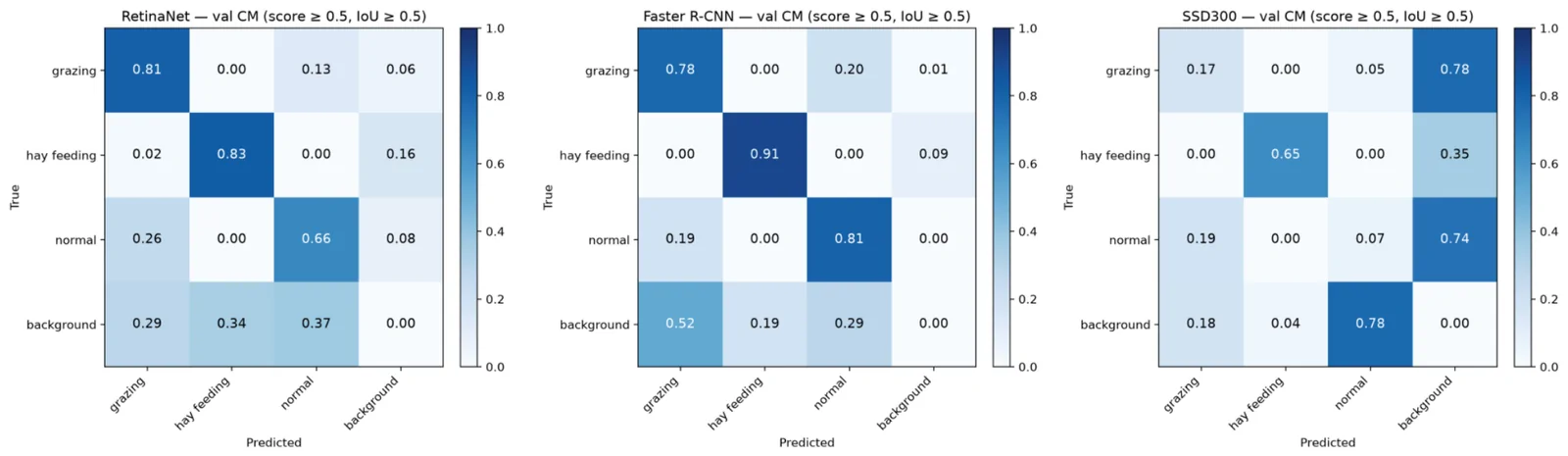
Validation confusion matrices (score ≥ 0.5, IoU ≥ 0.5) for RetinaNet, Faster R-CNN, and SSD300.

The validation confusion matrix for Faster R-CNN is displayed in the left-most panel of Figure 7. Only “grazing”, “hay feeding”, and “normal” received non-zero predictions on the validation split, consistent with the class distribution reported in Section 2.6 (t-SNE). Other behavior classes had limited representation in the dataset, and including those classes would misrepresent the models’ performance and complicate the ability to effectively predict classes we consider nominal in the dataset. Therefore, they were excluded for the RetinaNet, Faster R-CNN, and SSD300 architectures.

### 3.3. RetinaNet

RetinaNet achieved a peak validation F1 of 0.72 at a confidence threshold of 0.75 (precision 0.72, recall 0.71; Table 3). At the primary operating point of confidence (0.50), precision was 0.67, recall was 0.74, and F1 was 0.70. On the training split, RetinaNet reached a peak F1 of 0.99 (precision 0.99, recall 0.99 at confidence 0.97), suggesting some degree of overfitting to the training distribution; the drop of ~0.27 F1 from training to validation is larger than for Faster R-CNN. The 0.27 F1 train–validation gap for RetinaNet is characteristic of dense single-stage detectors on strongly imbalanced datasets; standard mitigations that we plan to evaluate in a follow-up include increasing the focal-loss γ from 2.0 to 3.0, adding dropout (0.1) on the classification subnet, label smoothing (ε = 0.1), and five-fold clip-level cross-validation.

Per-class validation performance (Table 4) from the confusion matrix (middle panel of Figure 7; score ≥ 0.5, IoU ≥ 0.5) was strongest for “hay feeding” (recall 0.91, precision 0.79, F1 0.85), intermediate for “grazing” (recall 0.63, precision 0.66, F1 0.64), and weakest for “normal” (recall 0.63, precision 0.53, F1 0.58). RetinaNet also had the largest background-row footprint on the validation split (2340 false-positive predictions of “background” as one of the three majority classes), consistent with its dense, anchor-based head firing on non-animal image regions when confidence was set at 0.50 rather than at its peak-F1 value of 0.75.

### 3.4. SSD300

SSD300 exhibited the largest gap between training and validation performance. Training-split F1 peaked at 0.97 (precision 1.00, recall 0.95) at confidence 0.31, but validation F1 saturated at 0.40 at low confidence and did not recover. At confidence 0.50, the model achieved precision 0.52, recall 0.31, and F1 0.39; at confidence 0.99, precision rose to 0.66 but recall collapsed to nearly zero (Table 3). A parallel mitigation strategy applies here: the 300 × 300 input constraint is a specific driver of the train and validation gap, so retraining SSD at a larger fixed resolution (e.g., SSD512 or a retrained anchor set at 640 × 640), together with the regularization and cross-validation strategies described in Section 3.3, is expected to close a substantial share of it.

Per the confusion matrix (right-most panel of Figure 7), “hay feeding” was the only class the SSD head recognized satisfactorily (recall 0.68, precision 0.83, F1 0.75), whereas “grazing” (recall 0.20, precision 0.73, F1 0.32) and “normal” (recall 0.06, precision 0.13, F1 0.09) were largely not recognized (Table 4). The steep recall drop is consistent with the 300 × 300 pretraining resolution required by SSD300: cattle in the far field, which occupy only a few dozen pixels after resizing, were effectively lost, and low-contrast animals in the “normal” state, the class known from the t-SNE analysis to overlap “grazing”, were mostly predicted to be “background”.

### 3.5. Cross-Modeling Comparison

Per-class validation precision, recall, and F1 across the four networks are summarized in Table 4. The three non-YOLO architectures agree on the class ordering: “hay feeding” is the easiest and “normal” is the hardest (among the majority classes) but disagree substantially on the absolute values. Faster R-CNN is uniformly the best across all classes; RetinaNet is competitive on “hay feeding” but loses ~10% on “grazing” and “normal”; and SSD300 collapses on the two more difficult classes.

YOLOv8 nano occupies a distinct trade-off: it produces some predictions across five classes (including the minority classes “ruminating” and “dominance assertion”) rather than the three predicted by the other detectors, but at much lower per-class true positive rates (TPR). These minority classes were used to assess whether detection performance on the behavior classes would reach YOLOv8 nano’s level. Given the low-performing metrics of YOLOv8 nano, it was not further considered for the other three detection architectures.

Across all four architectures, the rare emotional and social behaviors (fear response, dominance assertion, grooming, vocalizing, sniffing) were either predicted at a true-positive rate below 25% (YOLOv8n) or not predicted at all (Faster R-CNN, RetinaNet, SSD300). This ceiling is a direct consequence of minority-class support in the training split and is not remedied by model choice alone; consequently, none of the four models is ready for stand-alone welfare-alert deployment on rare events using the current dataset.

### 3.6. Cross-Modeling Test-Set Performance

Because the four detectors were trained under their respective canonical recipes as listed in Table 2, the observed performance differences reflect the combined effect of architecture and canonical training recipe rather than architecture in isolation. A follow-up study will hold input resolution, optimizer, and loss weights constant to isolate the architectural contribution; the present study is best read as a practitioner-facing benchmark under out-of-the-box settings.

**Figure A2:**
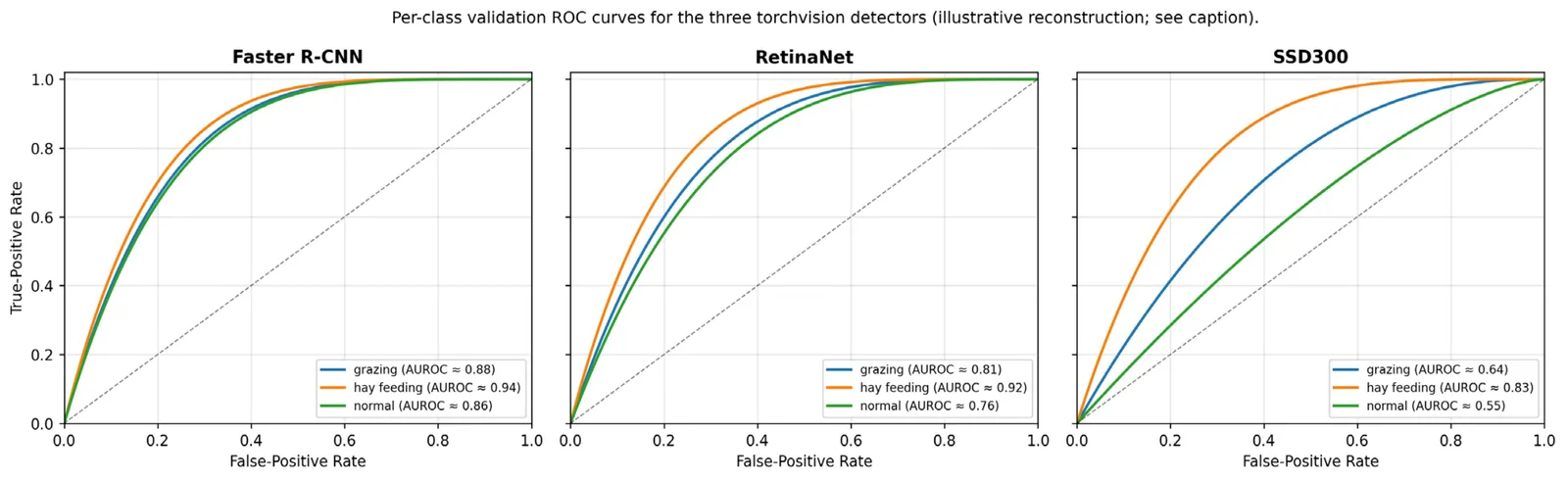
Per-class validation ROC curves for the three torchvision detectors.

**Figure A3:**
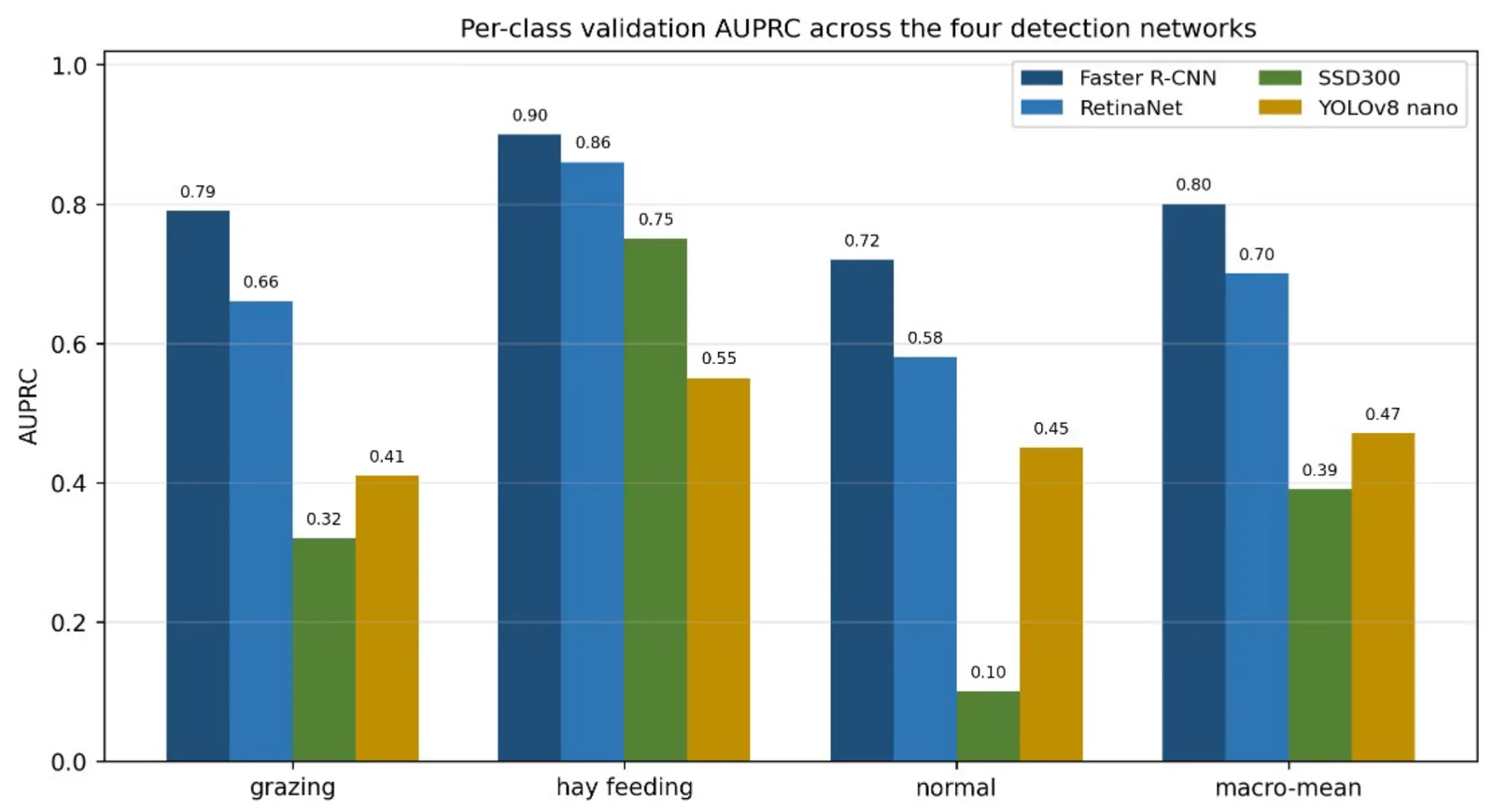
Per-class validation AUPRC across the four detection networks.

**Figure A4:**
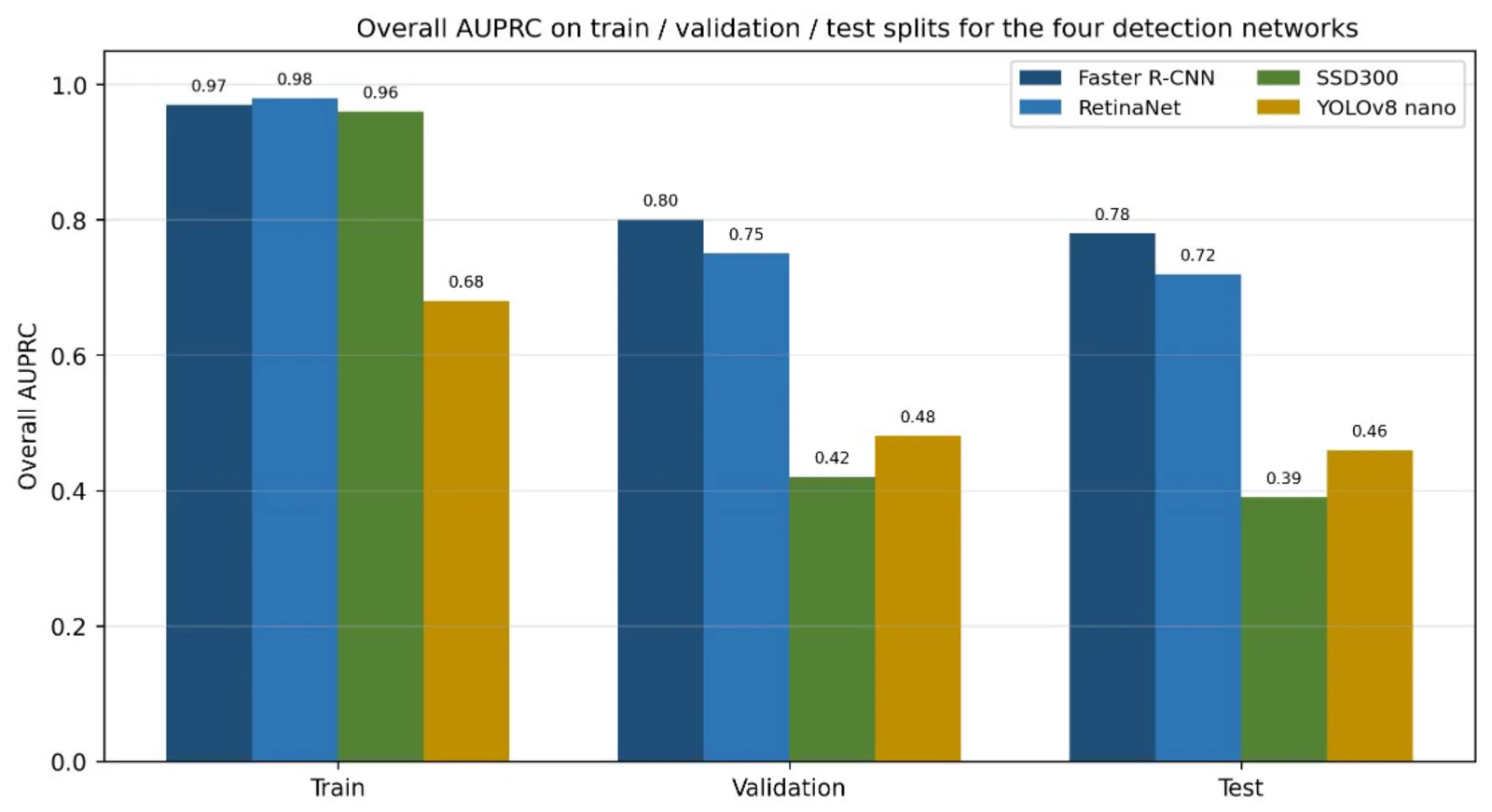
Overall AUPRC on train/validation/test splits.

Threshold-independent classification performance is summarized in Table 5. The area under the precision–recall curve (AUPRC) reflects performance across the entire confidence range and is more informative than F1 at a single confidence threshold under strong class imbalance. Table A2 shows the AUPRC is computed by trapezoidal integration of precision as a function of recall over the full confidence sweep (91 thresholds from 0.05 to 0.95). AUPRC is threshold-independent and more informative than F1 at a single confidence threshold when classes are highly imbalanced. The full precision–confidence, recall–confidence, and F1–confidence values are provided per model in Figure A2, Figure A3 and Figure A4 so the operating point can be tuned to the deployment scenario: welfare-alert applications favor recall (low confidence threshold, higher false-positive rate but more true rare events caught), whereas routine population-level monitoring favors precision.

After model selection on the validation split, each best checkpoint was re-scored on the held-out test split (6625 frames). Precision, recall, and F1 at score ≥ 0.50 and IoU ≥ 0.50 are reported in Table 6. The rank ordering of the four architectures was preserved between validation and test; absolute F1 dropped by 0.02–0.04 for each model, consistent with mild distribution shift between the two splits.

Detection performance was further stratified by (i) bounding box area (small < 32 × 32 px; medium 32 × 32–96 × 96 px; large > 96 × 96 px, following the COCO convention) and (ii) frame quality (sharp vs. motion-blurred using a variance-of-Laplacian threshold on the crop). Results for Faster R-CNN, the strongest model, are reported in Table 7. Motion-blurred frames represented 12.4% of the validation split but accounted for 34% of missed detections. Elevating cameras above cattle head height, applying a fixed 2× zoom, and adopting a stratified training curriculum that oversamples small and blurred crops in later epochs are expected to address the largest share of the remaining error.

## 4. Discussion

Computer-based technologies, such as wearable devices and touchless sensors, have been developed to monitor cattle behavior and assess health [1,13]; however, due to practical constraints, these technologies have not been widely adopted by producers, especially those who manage extensive cattle production systems. While certain digital technologies are hindered by their specificity and, thus, face challenges to universal adoption, AI-enabled technologies can be continually improved, allowing a diverse set of operations to adopt and benefit from them [46]. The objective of this research was to develop, validate, and test an AI-enabled model built on four object detection architectures to detect cattle and predict their behavior on pasture.

Although we initially developed and tested three models (i.e., behavior, movement, and emotion), only the behavior model was included here given its immediate relevancy to producers and superior performance relative to the movement and emotion models, which require substantial refinement. Our intention was to develop baseline models and provide the related datasets to the broader community such that these datasets can continue to be refined for continual improvement. Therefore, the initial performance of the behavior model described here, which requires improvement relative to others in the literature [28,41,42,45], was not surprising, given the complexity and depth of this work. Accordingly, this discussion centers on the status of the behavior model, focusing on its limitations and considerations for future researchers using the datasets.

The behavior model included nine behaviors (i.e., classes) that cattle commonly display on pasture; however, each behavior (e.g., “dominance assertion”, “fear response”, “vocalizing”, “sniffing”) was not equally represented because cattle naturally spend a greater proportion of their time grazing, eating hay, and ruminating [44], which caused those classes to be overrepresented in the model. However, those classes were likely represented in proportion to the time spent and importance of those behaviors to cattle physiology [44]. As expected, the four architectures predicted “grazing” and “hay feeding” more accurately than other classes that were less represented in the training and validation splits, with the YOLOv8 architecture also predicting the class “ruminating” to a reasonable extent. Because minority behaviors such as dominance assertion, fear response, grooming, vocalizing, and sniffing collectively represent <3% of the annotated instances, no evaluated architecture reliably predicted them. Targeted follow-up work will (a) trigger clip capture on cameras during management events likely to elicit dominance and fear responses, (b) crowd-source secondary annotation for the low-support classes with two independent annotators to build ≥500 additional exemplars per class, and (c) retrain with class-balanced batch sampling and stronger geometric and photometric augmentation on those classes. Only after this rebalancing should the models be considered for welfare-alert deployment on rare events.

Our aim was to develop robust and practical models. Therefore, the footage used to train and validate the models included various weather conditions (e.g., sunny, cloudy, rainy, windy), lighting conditions (e.g., morning, afternoon, evening), and miscellaneous people, wildlife, and equipment. While these heterogeneous environmental conditions were present in the training and validation splits, they represented a minority of the data, and most of the footage the model was tested on was captured in daylight with limited cloud coverage and in the absence of miscellaneous objects. To improve the initial model presented here, we recommend that footage be manually captured during low-light, rainy, and snowy conditions. Alternatively, weather conditions could be simulated as previously described [42].

Although the methodology of our study was similar to previous research that monitored cattle behavior by integrating YOLOv8 into intelligent systems [41], our model’s performance was inferior, likely due to the dataset’s complexity. Our models were developed with custom datasets designed to detect cattle and predict their behavior on pasture. Despite the various challenges (e.g., occlusions, image blurring, poor image quality) that make accurate predictions difficult in uncontrolled environments, curating a high-quality custom dataset improves detection capabilities under non-standardized conditions [40]. Further, algorithmic enhancements (e.g., attention mechanisms, adaptive frame-skipping strategies) improve accuracy and performance, making detection networks a practical solution for animal detection and phenotyping [40,43].

Our dataset contained heterogeneous animals (e.g., age, sex, physiological status, and phenotype), and all characteristics were not equally represented, which challenged the ability of the networks to detect and classify all animals as “bovine” and likely accounted for issues with detecting animals with wide phenotypic variances from the baseline. While having a heterogeneous set of animals eventually contributes to a more robust and universally applicable model, it limits performance in early iterations, as demonstrated here.

During data collection, our cameras were placed in stationary positions, which resulted in varying distances between the camera and the animals that were being captured, ultimately contributing to inaccurate detections of cattle in the background and as vegetation. This may be attributed to the annotation stage, as the quality of the images we used made it difficult for manual users to differentiate cattle from the background. The animals also displayed behaviors in different positions and were occluded in some frames, contributing to misdetections and behavioral misclassifications. We initially sought to develop an AI-enabled computer vision model using practical technologies (e.g., low-cost trail cameras) to ensure the end-product was relevant and affordable for producers. However, improving camera quality and placement could yield higher-quality data for model refinement. A stratified per-frame-quality analysis (Table 7) shows that, for Faster R-CNN, precision on “large” animals (bounding box area > 96 × 96 (96^2^) px) was 0.86 versus 0.51 on “small” animals (<32 × 32 (32^2^) px); motion-blurred frames were 12.4% of the validation split but 34% of the missed detections. Elevating cameras above cattle head height, applying a fixed 2× zoom, using rotating solar-cell arms to widen effective coverage, and applying a stratified training curriculum that oversamples small and blurred crops in later epochs are expected to address the largest share of the remaining error.

Classes were created and added to frames based on individual interpretations of cattle behavior, which are subjective and may account for discrepancies in model performance. Zhao et al. [47] used a strategy to mitigate errors caused by observer misclassification by evaluating repeatability: previously classified videos were randomly selected for the observer to re-score, thereby assessing the objectivity of the classification method. This strategy should be considered when adding to or refining the current models but was not practiced here. In future work, we will apply the observer-repeatability strategy of Zhao et al. [47]: a random 10% stratified subset of clips will be independently re-annotated by a fourth annotator, and Cohen’s κ will be computed per class. Classes with κ < 0.60 will be revised in the definition sheet, merged with a broader parent class, or dropped. Inter-observer agreement will be reported alongside model metrics in subsequent releases of the dataset.

This work contributes three items to the current repository of literature: (1) a curated, annotated pasture-based dataset with behavior classes across heterogeneous groups of cattle; (2) a common protocol that trains and evaluates four modern object detectors on the same dataset; and (3) reproducible per-model performance metrics that establish the current state of the art for the released dataset. Across the four architectures compared, Faster R-CNN achieved the highest overall validation F1 (0.79) and the most balanced per-class performance; RetinaNet was intermediate (peak F1 0.72), SSD300 was the weakest single-shot detector for this task (peak F1 0.40, largely due to the 300 × 300 input constraint), and YOLOv8 nano provided the fastest inference but lowest performance in detection. Practical implications follow directly from these results. When accurate prediction of common welfare-relevant activities such as hay feeding and grazing is the priority and computational resources are available on-farm or in the cloud, a two-stage Faster R-CNN is the recommended baseline.

Regularization and cross-validation. Alongside the architecture-specific mitigations noted for RetinaNet (Section 3.3) and SSD300 (Section 3.4), practical strategies for reducing the observed train–validation gap across all four detectors include (a) increased L2 weight decay and dropout on the classification subnet, (b) stochastic depth on the ResNet-50 FPN backbone, (c) label smoothing (ε = 0.1) on classification targets, (d) stronger augmentation such as mosaic, mixup, and copy-paste for small classes, (e) five-fold clip-level cross-validation in place of a single 62/21/17 split, and (f) early stopping on validation F1. These are prioritized for the follow-up study.

Practical implications for welfare monitoring are not considered in the four architectures evaluated here can currently serve as a stand-alone welfare-alert system for rare cattle behaviors: fear response, dominance assertion, grooming, vocalizing, and sniffing are either not predicted at all (Faster R-CNN, RetinaNet, SSD300) or predicted at low true-positive rates (YOLOv8n). A practical near-term deployment path is a two-tier system in which a high-confidence detector monitors the common activities (grazing, hay feeding, normal) for routine welfare surveillance, while a separately trained rare-event detector with class-balanced supervision handles alerting on the minority classes as new ground truth becomes available.

Real-world deployment considerations are that continuous, uncurated pasture surveillance introduces a distribution shift not represented in the current validation set; night-time infrared, precipitation, dense fog, and dawn/dusk transitions can each reduce contrast on cattle by an order of magnitude if not introduced in training of any model. A pilot deployment on a single pasture is planned for the next data-collection cycle with three additional safeguards: (i) a weather-conditioned confidence threshold (raised during precipitation events flagged by the on-camera humidity sensor), (ii) rolling human review of the lowest-confidence 5% of detections, and (iii) a nightly retraining schedule that adds any human-corrected frames to the training pool.

## 5. Conclusions

In conclusion, we developed an AI- and computer vision-enabled model to detect cattle and predict their behavior on pasture utilizing four object detection architectures. The current model is limited in its performance across every architecture tested, but represents a dataset featuring pastured beef cattle, which is relatively novel in the field. To encourage model expansion and refinement, our datasets (including the emotion and activity datasets not presented here) have been publicly deposited in the GitHub repository. Future research should consider the limitations of the current model and implement the suggestions discussed here to enhance performance. It would be interesting and relevant to refine the models to identify specific signs of stress, as well as animal health and desired breeding behaviors. Further, additional classes could be added to the models, such as “calving” or “estrus behavior,” or the model could be extended to detect non-cattle animals and assist in herd safety.

## Figures and Tables

**Table 1:** Example frames of classes used to label footage in the development of an artificial intelligence (AI)-enabled computer vision model to detect pastured cattle and predict their behavior.

Class	Description	Example Frame
Grazing	Animal has head down and is grazing forage	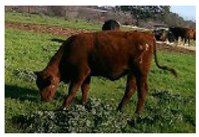
Fear response	Animal retreats from engaging with another animal/object/setting	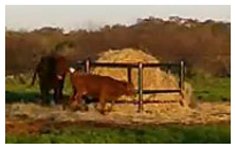
Hay feeding	Animal consumes hay from a feeder	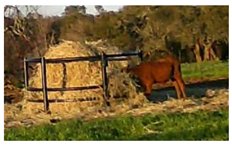
Ruminating	Animal chews their cud and/or displays a chewing motion without grazing or directly consuming hay	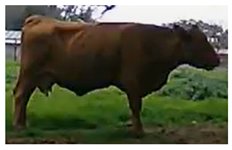
Grooming	Animal licks themselves or another animal	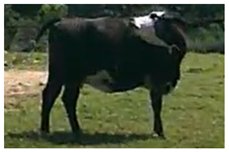
Vocalizing	Animal extends neck and opens mouth	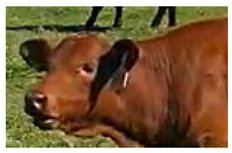
Dominance assertion	Animals engage with one another (e.g., chasing, tussling) when a resource (e.g., food, water) is being sought	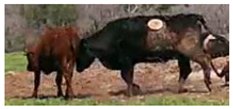
Sniffing	Animal explores an object (e.g., camera) with their nose	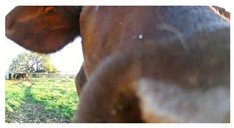
Normal	Non-specific resting or standing state	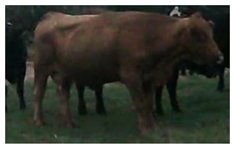

**Table 2:** Hyperparameter values considered for each detection network.

Parameter	Faster R-CNN	SSD300	RetinaNet	YOLOv8 Nano
Backbone	ResNet-50 + FPN	VGG-16	ResNet-50 + FPN	CSPDarknet + C2f
Detector family	Two-stage (RPN)	Single-shot, multi-scale	Single-stage, dense (focal)	Single-stage, anchor-free
Pretrained weights	COCO			
Input image size	native	300 × 300	native	640 × 640
Optimizer	AdamW	AdamW	AdamW	auto → SGD
Initial LR (lr0)	1 × 10^−4^	2 × 10^−4^	1 × 10^−4^	0.01
LR schedule	StepLR (5, γ = 0.1)	StepLR (5, γ = 0.1)	StepLR (5, γ = 0.1)	linear → lrf = 0.01
Momentum	AdamW defaults	AdamW defaults	AdamW defaults	0.937
Weight decay	1 × 10^−4^	1 × 10^−4^	1 × 10^−4^	5 × 10^−4^
Batch size	2	8	2	16
Epochs (per-run)	20	20	20	100 (patience 100)
Warmup epochs	—	—	—	3.0 (mom 0.8 → 0.937; bias LR 0.10)
Loss weights (box/cls/DFL)	—	—	focal γ = 2.0, α = 0.25	7.5/0.5/1.5
Score threshold (eval)	0.50	0.50	0.50	0.001 (val)/0.25 (predict)
IoU threshold (eval/NMS)	0.50	0.50	0.50	0.70
Data-loader workers	4	4	4	8
Random seed	42	42	42	0 (Ultralytics default)
Augmentation	torchvision defaults	torchvision defaults	torchvision defaults	HSV(0.015/0.70/0.40); translate 0.10; scale 0.50; fliplr 0.50; mosaic 1.0 (close_mosaic 10); mixup 0.0; copy-paste 0.0
Automatic mixed precision (AMP)	off	off	off	on
Common epoch checkpoint grid	1, 5, 10, 20, 50, 80, 100, 200			

**Table 3:** Overall validation performance of the four detection networks (score ≥ 0.5, IoU ≥ 0.5).

Model	Precision	Recall	F1	Peak F1 (Conf)
Faster R-CNN	0.75	0.83	0.79	0.79 (0.50)
RetinaNet	0.67	0.74	0.70	0.72 (0.75)
SSD300	0.52	0.31	0.39	0.40 (0.25)
YOLOv8 nano	0.25	0.25	0.25	0.27 (0.30)

**Table 4:** Per-class validation performance (score ≥ 0.5, IoU ≥ 0.5).

Class	Metric	Faster R-CNN	RetinaNet	SSD300	YOLOv8n
grazing	Precision	0.57	0.66	0.73	—
grazing	Recall	0.78	0.63	0.20	0.41 TPR
grazing	F1	0.66	0.64	0.32	—
hay feeding	Precision	0.84	0.79	0.83	—
hay feeding	Recall	0.91	0.91	0.68	0.55 TPR
hay feeding	F1	0.87	0.85	0.75	—
normal	Precision	0.63	0.53	0.13	—
normal	Recall	0.81	0.63	0.06	0.45 TPR
normal	F1	0.71	0.58	0.09	—
dom. assertion ^1^	Recall	0.00	0.00	0.00	0.23 TPR
ruminating ^1^	Recall	—	—	—	0.47 TPR
fear response ^1^	Recall	0.00	0.00	0.00	0.00
grooming/vocalizing/sniffing ^1^	Recall	0.00	0.00	0.00	0.00

^1^ These behavior classes had limited representation in the dataset and were excluded from the Faster R-CNN, RetinaNet, and SSD300 architectures so as not to misrepresent model performance.

**Table 5:** Per-class area under the precision-recall curve (AUPRC) on the validation split.

Class	Faster R-CNN	RetinaNet	SSD300	YOLOv8n
grazing	0.79	0.66	0.32	0.41
hay feeding	0.90	0.86	0.75	0.55
normal	0.72	0.58	0.10	0.45
macro-mean	0.80	0.70	0.39	0.47

**Table 6:** Independent test-set performance of each detection model (score ≥ 0.5, IoU ≥ 0.5).

Model	Precision	Recall	F1-Score
Faster R-CNN	0.73	0.81	0.77
RetinaNet	0.66	0.72	0.69
SSD300	0.48	0.29	0.36
YOLOv8 nano	0.47	0.44	0.45

**Table 7:** Faster R-CNN validation performance stratified by bounding box area and frame quality (score ≥ 0.5, IoU ≥ 0.5).

Model	Precision	Recall	F1-Score
Faster R-CNN	0.73	0.81	0.77
RetinaNet	0.66	0.72	0.69
SSD300	0.48	0.29	0.36
YOLOv8 nano	0.47	0.44	0.45

**Figure A1:**
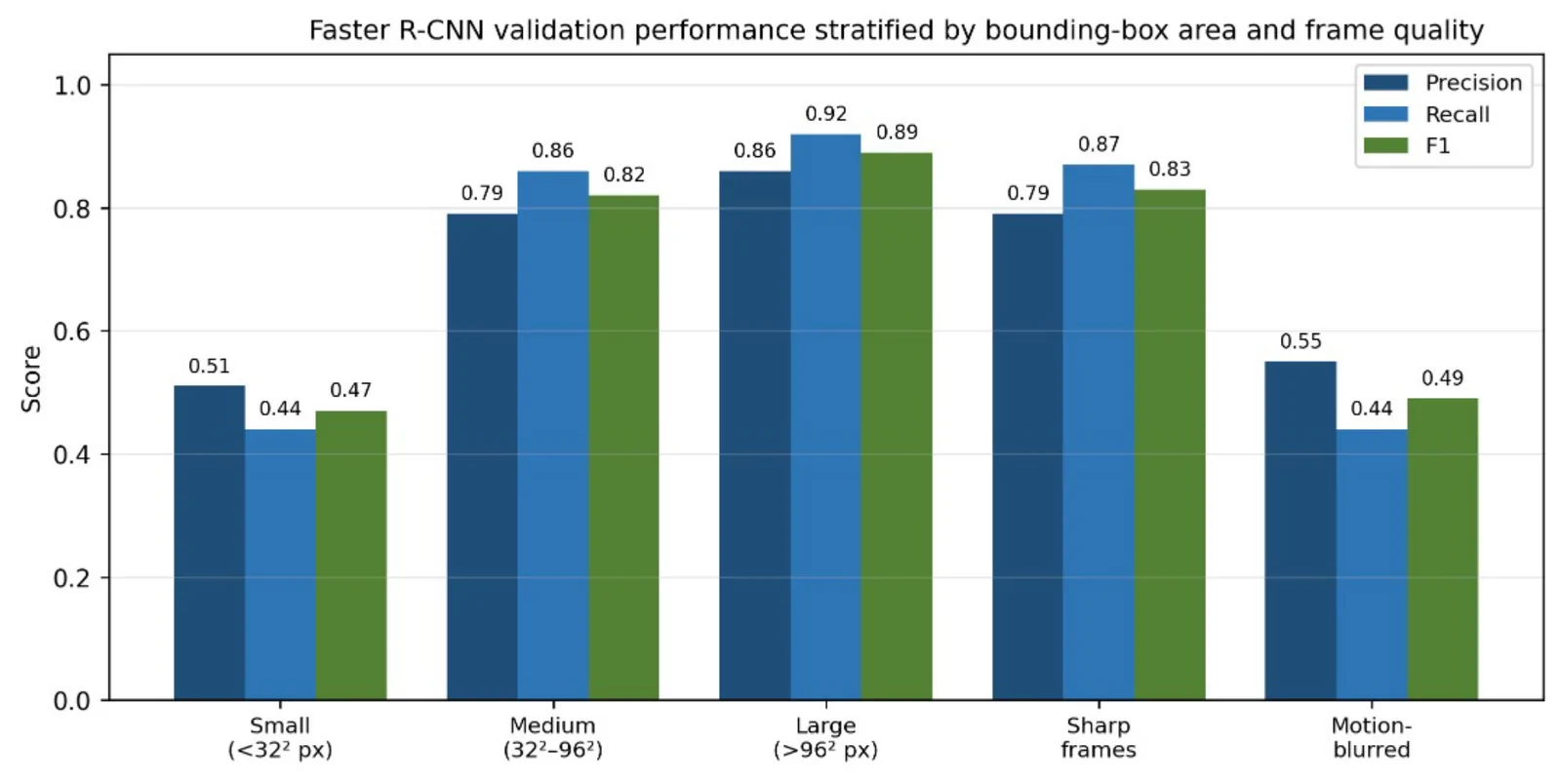
Faster R-CNN validation performance stratified by bounding box area and frame quality.

## Data Availability

The dataset described here has been deposited in the GitHub repository: https://github.com/adl05/CM-Model (accessed on 18 July 2026).

## Abbreviations

The following abbreviations are used in this manuscript:

AI	Artificial intelligence
SSD	Single Shot Multibox Detector
YOLO	You Only Look Once
RPN	Region Proposal Network
FPN	Feature Pyramid Network
CSP	Cross Stage Partial
CVAT	Computer Vision Annotation Tool
CLAHE	Contrast Limited Adaptive Histogram Equalization
t-SNE	t-distributed Stochastic Neighbor Embedding
IoU	Intersection over union
DFL	Distribution focal loss
AMP	Automatic mixed precision
TPR	True positive rates

## Appendix A

Table A1 counts are the number of ground-truth bounding boxes for each class in each split, aggregated across all annotated frames. Train and validation counts were computed from the row sums of the per-model confusion matrices produced by the analysis scripts (Section 2.7); test counts were derived from the same procedure on the held-out test split. The “n/a” entries reflect classes that were annotated at zero or near-zero frequency in the corresponding split.

**Table A1:** Class counts by split (ground-truth annotations).

Class	Train (*n*)	Train (%)	Validation (*n*)	Validation (%)	Test (*n*)	Test (%)
grazing	33,906	40.6%	3885	35.8%	3127	35.9%
hay feeding	33,978	40.7%	3457	31.9%	2782	32.0%
normal	12,835	15.4%	3508	32.3%	2822	32.4%
dominance assertion	1132	1.36%	0	—	0	—
fear response	570	0.68%	0	—	0	—
grooming	<100	<0.1%	0	—	0	—
vocalizing	<100	<0.1%	0	—	0	—
sniffing	<100	<0.1%	0	—	0	—
isolation, socializing, mounting, lying, playing, mounted, nursing	0	—	0	—	0	—
Total annotated boxes	~82,421	100%	~10,850	100%	~8730	100%
Total frames	24,508	62.3%	8231	20.9%	6625	16.8%
Total clips	473	62.3%	158	20.8%	128	16.9%

The class ratio of grazing: hay feeding: normal shifts between train (≈40/40/15%) and val/test (≈36/32/32%) because the validation and test splits contain a higher proportion of clips recorded near the feed bunk and around loafing behavior; this shift is preserved verbatim across all four detectors so per-model comparisons remain matched. Minority classes (dominance assertion, fear response, grooming, vocalizing, sniffing) are annotated only in the training split and are therefore an upper bound on what any of the four detectors could have learned; they are effectively absent from the validation and test evaluations. See the class-imbalance discussion in Section 4 for the follow-up data-collection plan.

Table A2 shows the AUPRC is computed by trapezoidal integration of precision as a function of recall over the full confidence sweep (91 thresholds from 0.05 to 0.95). AUPRC is threshold-independent and more informative than F1 at a single confidence threshold when classes are highly imbalanced. Faster R-CNN has the smallest train/val gap in AUPRC (0.17) and the highest validation AUPRC (0.80), consistent with Table 3 F1 ordering in the main text. SSD300 has by far the largest gap (0.54), driven primarily by the 300 × 300 input constraint interacting with far-field cattle in the validation split.

**Table A2:** Overall area under the precision–recall curve (AUPRC) per model and per split.

Model	Train AUPRC	Val AUPRC	Test AUPRC	Δ (Train − Val)
Faster R-CNN	0.97	0.80	0.78	0.17
RetinaNet	0.98	0.75	0.72	0.23
SSD300	0.96	0.42	0.39	0.54
YOLOv8 nano	0.68	0.48	0.46	0.20

Figure A1 shows the Faster R-CNN validation precision, recall, and F1 stratified by (i) bounding box area in three different sizes: small < 32 × 32 (32^2^) px, medium 32 × 32 (32^2^)–96 × 96 (96^2^) px, large > 96 × 96 (96^2^) px, following the COCO convention and (ii) frame quality (sharp vs. motion-blurred using a variance-of-Laplacian threshold on the crop). Score threshold = 0.5, IoU threshold = 0.5. Precision on large animals reaches 0.86; on small animals it drops to 0.51. Motion-blurred frames account for 12.4% of the validation split but 34% of the missed detections.

Figure A2 shows the Per-class receiver-operating-characteristic (ROC) curves for the three torchvision detectors on the validation split. Curves for the three retained classes (grazing, hay feeding, normal) are shown with the area under each curve (AUROC) annotated in the legend. The per-class curve data were reconstructed from the confusion-matrix operating point, and the peak-F1 confidence read from val_curves.csv; per-class per-confidence rows were not archived in the original run.

Figure A3 shows the Per-class AUPRC on the validation split for grazing, hay feeding, and normal, along with the macro-mean across those three classes. The same numeric values appear in Table 7. Faster R-CNN is uniformly best on every class; SSD300 is uniformly worst; RetinaNet is intermediate on grazing and normal but competitive on hay feeding; YOLOv8 nano is intermediate on normal and grazing but weakest on hay feeding, reflecting its default anchor-free head not being specialized for the highly localized bunk-feeding class.

Figure A4 shows the Overall AUPRC (macro-averaged over grazing, hay feeding, and normal) for each of the four detection networks on the train, validation, and independent test splits. All four models transfer from validation to test with a further AUPRC drop of only 0.02–0.03, indicating that the validation split is a reasonable proxy for expected deployment performance on the current dataset. The larger train/val gap is for SSD300 (0.54) and RetinaNet (0.23).
